# Decline of nucleotide excision repair capacity in aging *Caenorhabditis elegans*

**DOI:** 10.1186/gb-2007-8-5-r70

**Published:** 2007-05-01

**Authors:** Joel N Meyer, Windy A Boyd, Gregory A Azzam, Astrid C Haugen, Jonathan H Freedman, Bennett Van Houten

**Affiliations:** 1 Laboratory of Molecular Genetics, National Institute of Environmental Health Sciences, Alexander Drive, Research Triangle Park, NC 27709, USA; 2 Laboratory of Molecular Toxicology, National Institute of Environmental Health Sciences, Alexander Drive, Research Triangle Park, NC 27709, USA

## Abstract

**Background:**

*Caenorhabditis elegans *is an important model for the study of DNA damage and repair related processes such as aging, neurodegeneration, and carcinogenesis. However, DNA repair is poorly characterized in this organism. We adapted a quantitative polymerase chain reaction assay to characterize repair of DNA damage induced by ultraviolet type C (UVC) radiation in *C. elegans*, and then tested whether DNA repair rates were affected by age in adults.

**Results:**

UVC radiation induced lesions in young adult *C. elegans*, with a slope of 0.4 to 0.5 lesions per 10 kilobases of DNA per 100 J/m^2^, in both nuclear and mitochondrial targets. L1 and dauer larvae were more than fivefold more sensitive to lesion formation than were young adults. Nuclear repair kinetics in a well expressed nuclear gene were biphasic in nongravid adult nematodes: a faster, first order (half-life about 16 hours) phase lasting approximately 24 hours and resulting in removal of about 60% of the photoproducts was followed by a much slower phase. Repair in ten nuclear DNA regions was 15% and 50% higher in more actively transcribed regions in young and aging adults, respectively. Finally, repair was reduced by 30% to 50% in each of the ten nuclear regions in aging adults. However, this decrease in repair could not be explained by a reduction in expression of nucleotide excision repair genes, and we present a plausible mechanism, based on gene expression data, to account for this decrease.

**Conclusion:**

Repair of UVC-induced DNA damage in *C. elegans *is similar kinetically and genetically to repair in humans. Furthermore, this important repair process slows significantly in aging *C. elegans*, the first whole organism in which this question has been addressed.

## Abstract 2

Repair of UVC-induced DNA damage in *Caenorhabditis elegans *is similar kinetically and genetically to repair in humans, and it slows significantly in aging *C. elegans*.

## Background

*In vitro *assays, cell culture systems, and simple unicellular organisms continue to be crucial in elucidating mechanistic aspects of the formation and repair of DNA damage. However, the ability to study DNA damage, and especially its repair *in vivo*, is somewhat limited in metazoans. Studies in mouse models have been very informative, but they are also expensive and time consuming. *Caenorhabditis elegans *is a powerful system that is increasingly used to study many human conditions that are affected by DNA damage and repair, including carcinogenesis [1,2], neurodegenerative diseases [3,4], and aging [5,6]. Homologs of many human DNA genes are present in the *C. elegans *genome [7,8], suggesting that this simple multicellular eukaryote might be a good model for the study of DNA repair processes in higher eukaryotes. Furthermore, evidence is building that many of these genes are homologous in function as well as sequence; mutations or RNA interference (RNAi) knockdown of apparent DNA repair homologs have produced genotoxin-sensitive phenotypes [7,9-14], and RNAi screens for genes that protect against mutations have identified DNA repair gene homologs in *C. elegans *[15]. Finally, the molecular pathways that mediate cellular response to DNA damage, including apoptosis, are fairly well conserved between *C. elegans *and humans [16,17].

Although there are some studies of DNA repair in *C. elegans *(for review [18,19]), a simple, versatile assay that permits the study of gene-specific damage and repair in this organism has not been described. We adapted a quantitative polymerase chain reaction (QPCR)-based assay [20,21] to detect damage and repair of damage in the nuclear and mitochondrial genomes of *C. elegans*. Using this assay, we asked two questions: is the repair of DNA damage induced by ultraviolet type C (UVC; 254 nm) in *C. elegans *comparable to that observed in mammals; and are DNA repair rates different in young and aging populations of *C. elegans*?

In mammals, repair of UVC-induced DNA damage occurs through nucleotide excision repair (NER) [22,23]. NER is operative only in the nucleus, and it is responsible for the removal of a large number of structurally diverse bulky DNA lesions. NER consists of two distinct molecular pathways: global genomic repair (GGR), in which lesions present in any portion of the genome are detected and removed; and transcription-coupled repair (TCR), in which lesions are detected and subsequently removed when they block the progression of RNA polymerase II. If *C. elegans *homologs of mammalian NER genes function in a similar manner, then loss-of-function mutations in key NER genes would inhibit repair. Furthermore, the repair of highly transcribed regions of the nuclear genome should be faster than that of poorly or nontranscribed regions of the nuclear genome. We tested these predictions, and additionally characterized the kinetics of repair of a well-transcribed nuclear region in order to ask whether the repair kinetics are similar to those observed in mammalian cells in culture.

We also asked whether repair of UVC damage is less efficient in the nuclei of aging than in those of young adult *C. elegans*. There is evidence that nuclear genome integrity may be related to the aging process in mammals [24,25] and that repair rates decline in mammalian cells in culture [25,26]. However, very few *in vivo*, whole organism data have been reported that address this hypothesis [27]. Furthermore, there is little evidence to support the hypothesis that DNA repair capacity is related to age in *C. elegans*, despite the extensive use of this organism as a model for aging [5,6]. In this study, we observed a 30% to 50% decrease in DNA repair in aging *C. elegans *(assayed at 6 days after L4 molt, corresponding to 60% of the population's mean adult lifespan), and then performed gene expression profiling in young and aging adults to generate hypotheses to explain the mechanism of that decline.

## Results

### Exposure to UVC radiation causes similar, dose-dependent damage in the nuclear and mitochondrial genomes

We adapted a QPCR assay for analyzing gene-specific DNA damage and repair to *C. elegans*. The QPCR assay quantifies DNA damage by utilizing the ability of many DNA lesions to block or inhibit the progression of DNA polymerases [20]. Under quantitative conditions, PCR amplification of large (about 10 to 15 kilobases [kb]) regions of genomic DNA is reduced in damaged samples as compared with less damaged samples. This reduction in amplification can be converted to a lesion frequency by application of the Poisson distribution [28]. The use of PCR methodology permits the detection of nuclear and mitochondrial lesions in nanogram quantities of total genomic DNA.

**Figure 1:**
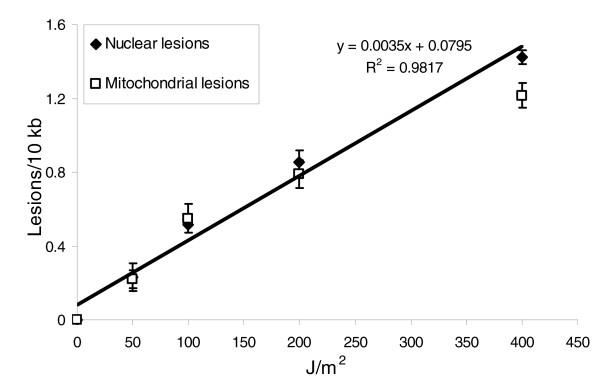
The nuclear and mitochondrial genomes exhibit similar lesion dose-responses after exposure to increasing UVC doses. The effect of dose was significant (*P *< 0.0001 for the main effect of dose), but the effect of genome was not, and neither did genome type alter the effect of dose (*P *= 0.4966 for main effect of genome, *P *= 0.9745 for dose × genome interaction) in a two-factor analysis of variance. *n *= 3-4 per point; error bars represent standard errors of the mean. UVC, UV type C.

Young adult (24 hours after L4 stage, hereafter referred to as '1-day-old') N2 (wild-type) nematodes exposed to 50, 100, 200, or 400 J/m^2 ^UVC (254 nm) irradiation exhibited a dose-dependent increase in lesions, as detected by QPCR (Figure 1). Lesions were induced with a slope of 0.4 to 0.5 lesions/10 kb per 100 J/m^2 ^UVC, with some loss of linearity evident at the higher doses. No difference was observed in lesion induction between nuclear and mitochondrial genomes. The nuclear target used was the DNA polymerase epsilon gene region; the mitochondrial target comprises the majority of the mitochondrial genome (see Materials and methods, below). Additionally, purified human and nematode genomic DNA were exposed to 5, 10, and 20 J/m^2 ^UVC, and damage quantified by QPCR using either previously described human primers (DNA polymerase beta [21]) or nematode DNA polymerase epsilon primers. The dose-response relation was indistinguishable for purified human and nematode genomic DNA (data not shown).

### Different life stages of *C. elegans *vary in susceptibility to UVC-induced nuclear and mitochondrial DNA damage

**Figure 2:**
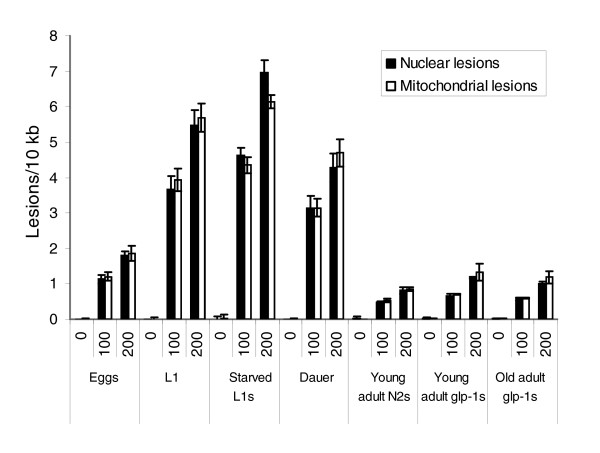
Marked variation in susceptibility to UVC-induced DNA damage in different life stages of *C elegans*. The UVC dose and life stage both had significant effects on induction of nuclear and mitochondrial lesions (*P *< 0.0001 for main effects of both), but no difference was observed between the nuclear and mitochondrial genomes (*P *= 0.9218 for main effect of genome) in a three-factor analysis of variance. All life stages and doses were statistically distinct from each other (*P *< 0.0001 in all cases, Fisher's protected least significant difference [FPLSD]), except the adult stages (*P *> 0.05 for all pair-wise comparisons, FPLSD). *n *= 3 for each column; error bars represent standard errors of the mean.

Different life stages of N2 or *glp-1 *nematodes exposed to 0, 100, or 200 J/m^2 ^UVC exhibited marked differences in susceptibility to induction of DNA damage (Figure 2), with starved L1 larvae the most and 1-day-old N2 adults the least susceptible. The *glp-1 *mutant is deficient in germline proliferation at 25°C [29], and only germline cells undergo division during adulthood in wild-type *C. elegans *[30]. The *glp-1 *mutant was used to permit unbiased study of DNA repair, as described below. These differences may relate to a size-related shielding effect, as addressed in the Discussion (see below). Again, no differences were observed in terms of damage to the nuclear (DNA polymerase epsilon target) and mitochondrial genome at any life stage. We also compared eggs isolated by bleach-sodium hydroxide treatment but not exposed to UVC, with unexposed eggs isolated by wash-off (eggs already laid), to test whether the bleach-sodium hydroxide treatment had a detectable effect on DNA integrity. No difference was detected (data not shown).

### Levels of DNA photoproducts decrease rapidly in UVC-exposed N2 and *glp-1 *adults

**Figure 3:**
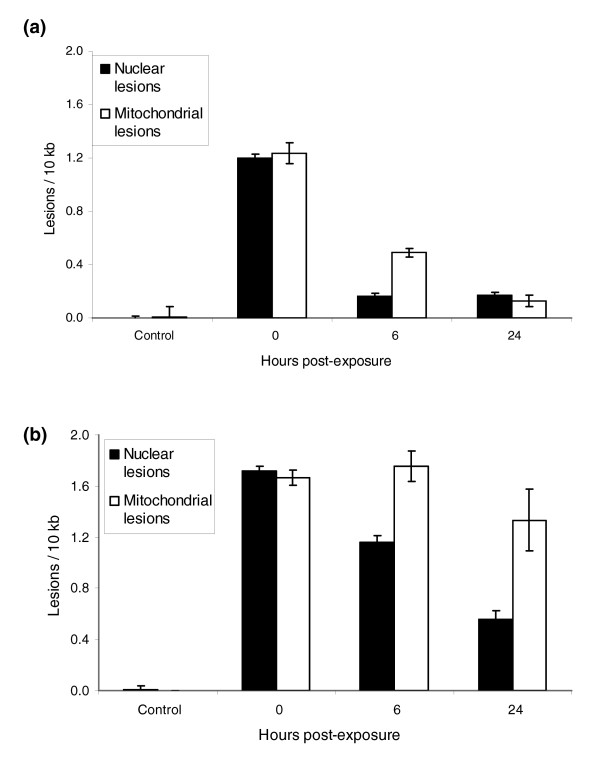
Disappearance of lesions from the nuclear and mitochondrial genomes of young adult nematodes. N2 data were not analyzed statistically, because they are unlikely to represent repair only (see text). For the *glp-1 *data, time had a significant effect on lesion frequency (*P *< 0.0001, main effect of time across genomes), but this effect was different for the two genomes (*P *= 0.0091, time × genome interaction). Time had a significant effect on lesion frequency when the nuclear lesion data were analyzed alone (*P *= 0.0004), but not when the mitochondrial data were analyzed alone (*P *= 0.068). *n *= 3 per column; error bars represent standard errors of the mean.

We exposed 1-day-old N2 adults to 400 J/m^2 ^UVC and either froze them immediately or after 6 or 24 hours of recovery. We measured DNA damage at each time point (controls plus 0, 6, and 24 hours after exposure). The lesion frequency decreased significantly (Figure 3a) in both the nuclear and mitochondrial genomes at 6 and 24 hours. However, young adult N2 nematodes are actively producing eggs, and so increases in amplification could be attributable either to repair of damaged DNA or to dilution of the initial pool of damaged DNA, with undamaged DNA produced during cell division. To eliminate the second possibility, we used 1-day-old *glp-1 *young adults raised at 25°C (N2 adults were also maintained at 25°C). Removal of nuclear lesions was apparent in *glp-1 *adults, whereas no statistically significant removal of mitochondrial lesions was observed (Figure 3b). The decrease in nuclear lesions observed in the *glp-1 *adults could not be attributed to cell division related dilution of damaged DNA, because there are no cell divisions in adult *glp-1 *mutants at 25°C [29]. Dead (defined as nonresponsive upon prodding) nematodes were not observed at any time point. Controls were frozen at the same time as the 0 hour recovery nematodes, because no change in background DNA lesions was observed over 24 hours in non-UVC-exposed nematodes. A somewhat higher level of initial lesions was observed in *glp-1 *than in N2 adults.

### Nuclear DNA repair is not detectably different in UVC-exposed *glp-1 *versus N2 starved L1 larvae

**Figure 4:**
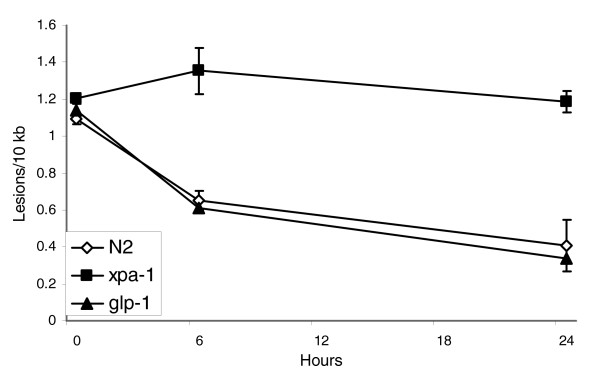
DNA repair in N2, *glp-1*, and *xpa-1 *starved L1 larvae. Repair was not detected in *xpa-1 *larvae, and not detectably different from wild-type N2 larvae in *glp-1 *larvae. Time and strain had a significant effect on lesion frequency (*P *= 0.007 and 0.003, main effects of time and strain, respectively), and the effect of time was different for different strains, indicating differential repair for some strains (*P *= 0.04, time × strain interaction). Among the different strains, *xpa-1 *was different from N2 and *glp-1 *(*P *= 0.002 in both cases) but N2 and *glp-1 *were not different from each other (*P *= 0.87). *n *= 2 to 3 per point; error bars represent standard errors of the mean.

To confirm that the difference in repair rate observed between *glp-1 *and N2 adults (Figure 3) was not due to an unexpected genetic difference in DNA repair rates, we exposed age-synchronized populations of *glp-1 *and N2 starved L1 larvae to 10 J/m^2 ^UVC, and measured lesion frequencies at 0, 6, and 24 hours after exposure. Because starved L1 larvae do not undergo cell division while they remain in the L1 stage, any decrease in lesion frequency in the nuclear target is attributable to DNA repair. No detectable difference in repair was observed between the N2 and *glp-1 *L1 larvae (Figure 4).

No deaths were observed in L1 larvae of any strain exposed to UVC, and neither were any L1 larvae observed to exit the L1 stage (no food was provided during the recovery period). Thus, the observed repair was not confounded either by death-associated DNA degradation or by cell division-associated DNA synthesis.

### Nuclear DNA repair is not detectable in UVC exposed *xpa-1 *starved L1 larvae

DNA repair in *xpa-1 *starved L1 larvae was not detected (Figure 4). XPA-1 is a homolog of the human xeroderma pigmentosum complementation group A protein, which plays a key role in the verification of DNA damage in the NER pathway [23]. The *xpa-1 *strain RB864 harbors a deletion of the last three exons of the *xpa-1 *gene, corresponding to about 80% of the protein. We verified the presence of the genomic deletion by PCR (data not shown) and carried out these experiments after out-crossing three times.

### Kinetics of nuclear DNA repair in UVC-exposed young adult *glp-1 *nematodes is biphasic

**Figure 5:**
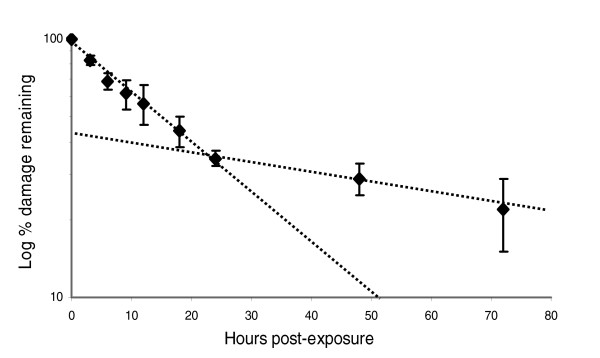
Kinetics of DNA repair in polymerase epsilon target in *glp-1 *adults following 400 J/m^2 ^UVC. The decrease in lesion frequency best fits first-order kinetics over the first 24 hours, but is slower after 24 hours. *n *= 3-8 per point; error bars represent standard errors of the mean.

Having established the suitability of the *glp-1 *strain for studies of DNA repair kinetics, we exposed 1-day-old *glp-1 *adults to 400 J/m^2 ^UVC, allowed them to recover for 3 hours to 3 days, and then analyzed lesion frequencies in the polymerase epsilon target (Figure 5). A semi-logarithmic plot of percentage lesions remaining versus time indicated biphasic repair; a rapid component lasting approximately 24 hours and characterized by a half-life of about 16 hours was followed by a much slower phase. Although dead nematodes were not observed, the UVC-exposed nematodes were sluggish between 24 and 72 hours after exposure. No change in background DNA lesions was observed in non-UVC-exposed nematodes over 72 hours.

### Highly transcribed nuclear genes are repaired more rapidly than poorly transcribed genes

We developed primers to amplify ten nuclear genes that were expected to be transcribed at one of three approximate levels in adults (Table 1 and Additional data file 1): not transcribed or very poorly transcribed (*n *= 4); transcribed at medium levels (*n *= 3); and transcribed at high levels (*n *= 3). These expectations, derived from literature and database values, were confirmed by gene expression profiling in the *glp-1 *strain and culture conditions used for studies of repair (Table 1). We then measured percentage repair in each of these targets in 1-day-old adult *glp-1 *nematodes at 25°C, 24 hours after exposure to 400 J/m^2 ^UVC. Despite the large differences in expression level of the target genes (Table 1), the differences in repair rates were relatively small in young adults: about 15% between poorly expressed and highly expressed genes. We also measured repair rates in the same ten nuclear targets in 6-day-old *glp-1 *adults (raised and maintained at 25°C) after exposure to 400 J/m^2 ^UVC. The difference in repair rates between genes was larger in aging than in young adults; highly expressed genes were repaired about 50% more quickly than poorly expressed genes in aging adults. The difference was statistically significant in both cases (*P *= 0.001 for young and *P *= 0.005 for aging adults; Table 1).

**Table 1:** Gene-specific repair in low, medium, and high expression genes in young and aging adult *glp-1 *nematodes

		Measured^b ^expression		Percentage repair after 24 hours	
		
Genomic target	Estimated^a ^transcription in adults	young adult *glp-1*s	aging adult *glp-1*s	young adult *glp-1*s	aging adult *glp-1*s
Low transcription genes					
*par-2 *(ubiquitin ligase; F58B6.3b)	None/low	10^a^	12^a^	61 ± 2	33 ± 10
*pax-3 *(homeodomain transcription factor; F27E5.2)	Low	2^a^	3^a^	49 ± 1	25 ± 4
*lin-39 *(HOX domain transcription factor; C07H6.7)	None/low	22^a^	48^a^	55 ± 1	30 ± 2
*nob-1 *(HOX domain transcription factor; Y75B8A.2)	None/low	12^a^	13^a^	60 ± 3	22 ± 2
Average				56 ± 3	28 ± 2
Medium transcription genes					
*unc-2 *(calcium channel α subunit; T02C5.5a)	Medium	19	42	55 ± 1	29 ± 8
polymerase epsilon (F33H2.5), F33H2.6, and part of *dog-1*	Medium	54	288	63 ± 5	32 ± 4
*atl-1 *(ATM-like protein kinase; T06E4.3)	Medium	88	146	70 ± 1	32 ± 1
Average				63 ± 4	31 ± 2
High transcription genes					
*act-1*, *act-2*, *act-3 *(T04C12.6, T04C12.5, T04C12.4), and about 3 kb noncoding	High	7,541	6,881	66 ± 2	39 ± 5
*unc-44 *(ankyrin; B0350.2a.1)	High	501	765	66 ± 4	48 ± 5
*act-4 *(M03F4.2), M03F4.6, and M03F4.7	High	4,018	3,946	63 ± 5	41 ± 3
Average				65 ± 1	42 ± 3

Shown are gene-specific repair in low, medium, and high expression genes in young (1 day after L4) and aging (6 days after L4) adult *glp-1 *nematodes. The decrease in repair with age is very highly significant when all genes are analyzed individually (effect of age across all genes, *P *< 0.0001). Different genes are repaired at different rates when analyzed individually (effect of gene analyzed, irrespective of age; *P *= 0.003; two-factor analysis of variance on effects of age and Affymetrix-derived expression level). When genes were grouped qualitatively for statistical analysis as 'low', 'medium', and 'high' transcription, the level of expression had a significant effect on repair rate in young (*P *= 0.001) and aging (*P *= 0.005) nematodes. In young adults, repair of 'low' expression genes was significantly different than 'medium' and 'high' expression genes by Fisher's protected least significant difference (FPLSD; *P *= 0.01 and 0.0006, respectively), but repair of 'medium' and 'high' expression genes was not different (*P *= 0.28). In aging adults, repair of 'high' expression genes was significantly different than 'medium' and 'low' expression genes by FPLSD (*P *= 0.02 and 0.001, respectively), but repair of 'medium' and 'low' expression genes was not different (*P *= 0.37). Percentage repair data are presented as mean ± standard error (*n *= 3-4 per gene per time point). Initial damage was comparable in 1-day and 6-day adults: 2.1 ± 0.25 lesions/10 kilobases (kb) in 1-day adults, and 2.0 ± 0.20 lesions/10 kb in 6-day adults. ^a^Estimated transcription levels were based on literature review. ^b^Measured expression data are average raw fluorescence values obtained from gene expression analysis in this study. ^c^Values flagged as 'absent' (below reliable detection). The variability in raw scores flagged as absent is due to variability in the measurements made from different chips. Raw data presented in this table were averaged when multiple probes were present for a specific mRNA, and weighted averages were used when more than one gene is included in the amplified target.

### Repair in nuclear genes is decreased in aging nematodes

**Figure 6:**
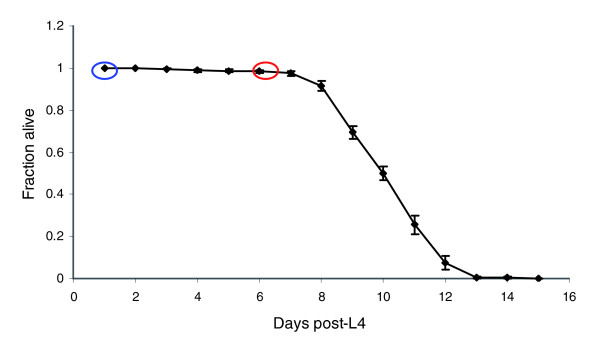
*glp-1 *adult lifespan at 25°C. One-day-old adults (1 day after L4 molt; time point circled in blue) are described in this paper as 'young' adults, whereas 6-day-old adults (6 days after L4 molt; time point circled in red) are described as 'aging' adults. One day after L4 molt was counted as 'day 1'; populations were maintained at 25°C from hatch. A total of eight replicate plates were monitored; three separate (in time) experiments were carried out. Additional details are given in the Materials and methods section (see text). Cuticular blisters were observed on 0 out of 100 randomly selected 1-day-old ('young') adults, but in 23 out of 100 randomly selected 6-day-old ('aging') adults; examples are shown in Additional data file 2. In Additional data file 2, hollow arrows point to tails without blisters, and solid arrows point to blisters. Mobility and feeding were markedly reduced beginning approximately on day 5, which was observable by inspection; the amount of OP50 eaten and distance traveled over 24 hours were also reduced. In 1-day-old ('young') adults, guts are cleared in about 30 min in M9 buffer; guts of 6-day-old ('aging') adults were not cleared, even after 2 hours.

Previous studies conducted in cells in culture have suggested that DNA repair declines with age in mammals [24,25]. We found that repair in all ten nuclear targets was lower in aging (6 days after L4) adults than repair of those same targets in young (1 day after L4) *glp-1 *adults (*P *< 0.0001; Table 1). This difference was greatest in low and medium expression genes (about 50% decrease) but was also robust in high expression genes (about 33% decrease). We chose day 6 to represent the aging adult population because at this age more than 98% of the population is still alive, but the population as a whole has reached 60% of its mean adult lifespan (10 days; Figure 6) and 43% of its maximum adult lifespan (14 days; Figure 6). One-day-old adults have reached 10% of the mean adult lifespan, and 7% of the maximum adult lifespan. *glp-1 *adults raised at 25°C exhibit signs of old age at 6 days, including constipation, cuticular blisters, and reduced mobility and feeding, but they have not yet begun to die in significant numbers (Figure 6 and Additional data file 2). It is therefore unlikely that repair rates are significantly confounded by DNA degradation occurring in dead animals. Initial lesion frequencies were not significantly different between young and aging adults (Table 1).

### Gene expression analysis reveals multiple changes in aging *glp-1 *adults

We used gene expression profiling to address several specific questions, as well as to generate hypotheses regarding the possible mechanism(s) of decreased DNA repair in aging *glp-1 *adults. The raw data are accessible at National Center for Biotechnology Information's Gene Expression Omnibus (GEO; accessible through GEO series accession number GSE4766), as are *p *values from Rosetta Resolver for all pairwise comparisons for all genes.

First, we confirmed that the transcriptional status of the nuclear genes utilized as targets to measure repair (see Materials and methods, below) were approximately what we had predicted (Table 1). Second, we found that the mRNA levels of all of those genes remained approximately constant between days 1 and 6 of adulthood in the *glp-1 *adults (Table 1), although a gene that is adjacent to the polymerase epsilon gene, and part of the amplified target (F33H2.6), increased by fivefold to sixfold in expression. Third, none of the NER gene homologs present in *C. elegans *[8] were expressed at lower levels in aging than in young adult *glp-1 *nematodes (*P *> 0.001 in all cases by Rosetta Resolver). Rather, these genes exhibited a general pattern of high expression in embryos, and then lower expression in both young and aging adults (Table 2). Two potentially important exceptions to this general pattern appeared to be Y50D7A.2 and *csb-1*, the *C. elegans *homologs of XPD (xeroderma pigmentosum complementation group D) and CSB (Cockayne Syndrome complementation group B). These genes were apparently expressed at lower levels in 6-day-old than in 1-day-old adults, although this difference was not statistically significant, and both had very low or below reliable detection signals on the arrays in the adult samples (Table 2). Because reverse transcription (RT)-PCR failed to confirm decreased expression of either gene in 6-day-old adults (data not shown), we do not believe that the decline in repair capacity with age is due to a decrease in either of these two gene products.

**Table 2:** mRNA ratios for NER and NER-related genes in embryonic, young, and aging adult *glp-1 *nematodes raised at 25°C

		Normalized expression values		
		
Human NER and NER-related genes	*C elegans *homologs	Embryonic *glp-1*s	Young adult *glp-1*s	Aging adult *glp-1*s
XPC	Y76B12C.2	5.589 (4.369 to 6.619)^a^	0.937 (0.621 to 1.491)^b^	1.526 (1.347 to 1.729)
RAD23A/B	ZK20.3	1.109 (1.024 to 1.212)	0.984 (0.776 to 1.204)	1.057 (1.01 to 1.106)
CETN2	T21H3.3	0.949 (0.76 to 1.125)	0.945 (0.591 to 1.357)	1.423 (1.244 to 1.628)
XPA	K07G5.2	3.107 (2.701 to 3.446)^a^	0.918 (0.585 to 1.576)	1.42 (1.33 to 1.516)
RPA1	F18A1.5	8.712 (7.521 to 10.17)^a^	0.995 (0.903 to 1.136)	1.701 (1.633 to 1.772)
RPA2	M04F3.1	4.133 (3.253 to 4.985)^a^	0.984 (0.769 to 1.179)	0.963 (0.743 to 1.247)
ERCC3 (XPB)	Y66D12A.15	1.352 (1.286 to 1.458)	0.994 (0.856 to 1.119)	1.789 (1.539 to 2.08)
ERCC2 (XPD)	Y50D7A.2	1.47 (1.262 to 1.865)	0.976 (0.71 to 1.203)	0.281 (0.279 to 0.283)^b^
GTF2H1	R02D3.3	3.791 (3.502 to 4.171)^a^	0.972 (0.741 to 1.325)	1.016 (0.915 to 1.128)
GTF2H2	T16H12.4	5.594 (4.509 to 6.348)^a^	0.973 (0.746 to 1.319)^b^	1.444 (0.977 to 2.135)
GTF2H3	ZK1128.4	1.326 (1.193 to 1.464)	0.917 (0.653 to 1.615)	1.611 (1.361 to 1.906)
GTF2H4	Y73F8A.24	1.78 (1.704 to 1.891)	0.955 (0.716 to 1.44)	1.304 (1.13 to 1.506)
GTF2H5 (TTDA)	Y55B1AL.2	0.917 (0.733 to 1.083)	0.929 (0.598 to 1.521)	0.839 (0.783 to 0.898)
CDK7	Y39G10AL.3	3.504 (3.173 to 4.126)^a^	0.967 (0.79 to 1.383)	1.467 (1.389 to 1.55)
CCNH	Y49F6B.1	23.67 (13.54 to 36.68)^a^	0.963 (0.727 to 1.386)^b^	1.553 (1.245 to 1.938)^b^
MNAT1	F53G2.7	1.563 (1.521 to 1.639)	0.884 (0.597 to 1.735)	1.9 (1.839 to 1.963)
ERCC5 (XPG)	F57B10.6	1.073 (1.02 to 1.132)	0.988 (0.796 to 1.146)	1.149 (1.056 to 1.251)
ERCC1	F10G8.7	1.018 (0.852 to 1.243)	0.957 (0.618 to 1.209)	0.857 (0.855 to 0.859)
ERCC4 (XPF)	C47D12.8	2.836 (2.41 to 3.451)^a^	0.992 (0.826 to 1.097)^b^	1.06 (0.909 to 1.237)^b^
LIG1	C29A12.3a	18.19 (14.65 to 23.2)^a^	0.997 (0.904 to 1.084)	0.752 (0.687 to 0.823)
CKN1 (ERCC8)	K07A1.12	15.04 (10.95 to 18.83)^a^	0.971 (0.707 to 1.282)	1.558 (1.269 to 1.911)
ERCC6 (CSB)	F53H4.1	0.479 (0.425 to 0.587)	0.995 (0.874 to 1.128)^b^	0.57 (0.551 to 0.589)^b^
XAB2 (HCNP)	C50F2.3	7.011 (5.565 to 8.512)^a^	0.993 (0.901 to 1.172)	1.511 (1.494 to 1.527)
DDB1	M18.5	1.499 (1.365 to 1.596)	0.993 (0.841 to 1.115)	1.768 (1.661 to 1.883)
DDB2	C18E3.5	6.884 (5.14 to 8.339)^a^	0.989 (0.851 to 1.203)^b^	1.419 (1.419 to 1.42)
TFF2	T23H2.3a	1.785 (1.298 to 2.093)	0.94 (0.66 to 1.505)^b^	0.907 (0.778 to 1.057)
MMS19L (MMS19)	C24G6.3	1.271 (0.947 to 1.72)	0.996 (0.887 to 1.084)	1.214 (1.024 to 1.439)

Shown are mRNA ratios for nucleotide excision repair (NER) and NER-related genes in embryonic, young (1 day after L4 molt), and aging (6 days after L4 molt) adult *glp-1 *nematodes raised at 25°C. ^a^Values that are significantly different (*P *< 0.001 by Rosetta Resolver) for embryos compared with young adults. No statistically significant differences between young and aging adults occurred. ^b^Raw fluorescence signals flagged by GeneSpring as absent (below reliable detection).

**Figure 7:**
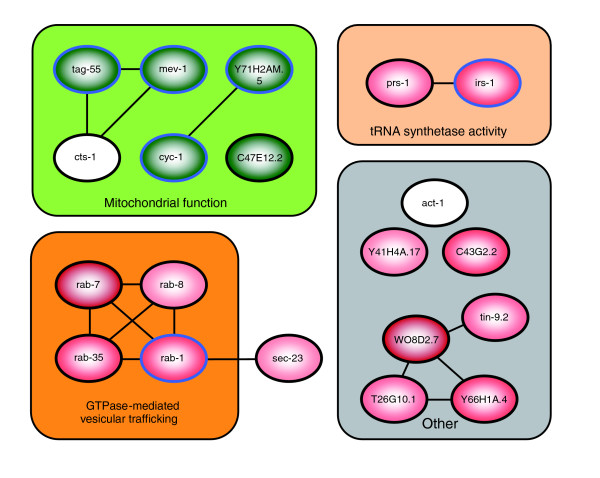
Top 20 central nodes representing perturbed neighborhoods, identified by Cytoscape. Green indicates downregulation, and red indicates upregulation of the gene/node in aging (6 days after L4) versus young (1 day after L4) *glp-1 *nematodes; darker shades indicate greater alteration. Blue borders indicate genes that were significantly different in expression individually in aging compared with young adults (*P *< 0.05). Nodes that are grouped into the gray cluster do not fall into a common Gene Ontology. Additional data file 4 (parts A and B) provides additional information about each of the nodes (genes) and the associated neighborhoods.

Because the initial hypothesis that decreased repair of UVC DNA damage could be explained by decreased transcription of NER genes was not supported, we used three bioinformatics programs to carry out higher level analysis of gene expression data: Cytoscape [31], GOMiner [32], and GeneSpring (Silicon Genetics; Gene Ontology [GO] and Kyoto Encyclopedia of Genes and Genomes [KEGG] functions). We used multiple programs based on different bioinformatics and statistical approaches to compensate in part for the incomplete nature of current *C. elegans *GO annotations and interactomes. A partial list of Gene Ontologies that were identified as important in at least two of the three bioinformatics approaches is presented in Table 3. Overlaying our gene expression data onto an interactome consisting of 4,669 nodes connected by 23,785 edges (see Materials and methods, below), and using the jActiveModules plugin for Cytoscape [31], we identified the top-scoring 20 nodes (genes) representing perturbed neighborhoods (subnetworks of interacting genes and proteins). These nodes, grouped when possible by high-level GO terms, are presented in Figure 7.

**Table 3:** Major biologic functions altered in aging versus young *glp-1 *adults

Biologic process/molecular function^a^	mRNA levels in aging versus young adult *glp-1*s	Proportion changed^b^	*P *value^b^
Larval development	Decreased	112/842	<0.0001
Ion transport	Decreased	113/504	<0.0001
Generation of precursor metabolites and energy	Decreased	78/474	<0.0001
Structural constituents of cuticle	Decreased	88/144	<0.0001
Lipid metabolism	Decreased	24/145	<0.0001
Oxidative phosphorylation	Decreased	13/44	<0.0001
Aging (*C elegans*)	Decreased	12/58	0.003
Catalytic activity	Decreased	215/2734	0.0004
Glycolysis	Decreased	4/15	0.014
Positive regulation of growth	Increased	124/928	<0.0001
Cytoskeleton	Increased	21/108	0.0003
Rab-related GTPase protein-mediated vesicular trafficking	Increased	16/72	0.0003
Aging (*C elegans*)	Increased	10/58	0.004
Osmoregulation	Increased	21/137	0.007
Locomotion	Increased	24/144	0.001

Shown are major biologic functions altered in aging (6-day) as compared with young (1-day) *glp-1 *adults. ^a^Listed Gene Ontologies were identified as different in young and aging *glp-1*s in at least two of three different bioinformatic analyses. ^b^Proportion changed and *p *values are as determined by GOMiner. Complete Gene Ontology analyses are given in Additional data files 3, 4 (parts A and B), and 5. I changed the format slightly to avoid breaking up "*glp-1*s".

More detailed results of the Cytoscape and GOMiner analyses are available in Additional data files 3 to 5. The top-scoring Gene Ontologies identified by GOMiner are available in additional data file 3. The top 20 central nodes depicted in Figure 7 are listed, along with gene descriptions and GO terms for each node, in Additional data file 4 (part A). The significant GO terms associated with all genes (to a depth of two neighbors) in each of the perturbed neighborhoods (Active Modules), as identified by the BiNGO plugin for Cytoscape [33], are listed in Additional data file 4 (part B). Finally, the top-scoring Gene Ontologies identified across the entire dataset (without first selecting Active Modules) by the BiNGO plugin for Cytoscape are listed in Additional data file 5. Our findings suggest a decrease in many processes that are fundamental to homeostasis, including ion transport, catalytic activity, and energy production. As addressed in the Discussion (below), the evidence that mitochondrial function is diminished in aging nematodes is particularly interesting.

## Discussion

We have found that the DNA repair process of NER is similar in *C. elegans *and humans in many important respects. Repair in *C. elegans *is comparable kinetically to mammalian repair [20,28], and occurs more quickly in transcribed than in nontranscribed genes. The relationship between repair rate and transcriptional status was strongest in aging adults. Repair was robust even in *glp-1 *adults, in which all cells are terminally differentiated. Additionally, we found that DNA repair is 30% to 50% slower in aging than in young adult nematodes. This finding extends previous findings of age-related decreases in repair capacity, made in mammalian cell culture studies, to a whole organism model. This suggests that an age-related decline in DNA repair is a common biologic phenomenon *in vivo*. Gene expression analysis did not support the hypothesis that decreased repair in aging adults is the result of decreased expression of DNA repair genes, but rather suggested the hypothesis that energy becomes limiting for DNA repair in aging worms.

### Characterization of UVC-induced DNA damage

Using the QPCR assay, we quantified a dose-dependent increase in nuclear and mitochondrial lesions in populations of *C. elegans *at various life stages (Figures 1 and 2). L1 larvae and dauer larvae were the most susceptible to DNA damage of the stages tested, and adults were the least. We speculate that smaller life stages may be less able to shield DNA from UVC radiation. It may be that the slightly increased lesion formation in *glp-1 *than in N2 adults (Figures 2 and 3) is due to shielding as well; N2 adults are wider because of the presence of many dividing germ cells and developing ooctyes. The chorion of the oocytes may also provide some shielding. Previous studies have provided evidence both against [34] and for [35-37] a significant effect of shielding. In the only previous study in which damage was directly measured in *C. elegans*, a difference was observed, although the pattern of the difference was not identical to the one that we observed; rather, lesion induction declined about 30% throughout development (embryos to young adult stages) [36]. Shielding may also explain the fact that approximately tenfold more UVC exposure is necessary to generate a given level of lesions in *C. elegans *adults than in human cells assayed using the same QPCR method (for instance, see Van Houten and coworkers [20]). It is well established that *C. elegans *is remarkably resistant to the toxic effects of UVC exposure [34,38-40], and 300 J/m^2 ^has been used for generation of mutants and transgene integration [41,42]. Although this UVC resistance may be partly explained by other phenomena such as high translesion synthesis [37], our results suggest that it is at least in part due to the fact that an equivalent amount of UVC simply produces fewer lesions in intact nematodes than in cells in culture.

Hartman and coworkers [37], the only other group we are aware of that has directly measured DNA damage in *C. elegans*, found (using the enzyme-sensitive site assay) that embryos exposed to UVC had about 0.5 cyclobutane pyrimidine dimers (CPDs)/10^8 ^daltons per J/m^2^. Assuming that 70% of the lesions measured by our assay are CPDs [43,44], we measured about 0.2 CPDs/10^8 ^daltons per J/m^2 ^(Figure 2). Although the calculated lesion frequencies are not identical, they are remarkably close, given that very different methods were used. The QPCR assay was sensitive enough to detect damage in nematodes exposed to levels of UVC more than an order of magnitude under lethal levels, and can be performed with total quantities of DNA much smaller than those required by most other DNA damage assays.

### Removal of UVC-induced DNA damage in wild-type and mutant adults and starved L1 larvae

The QPCR assay can be used to measure changes in lesion frequency over time, thus potentially quantifying repair of DNA after a single DNA-damaging event such as UV exposure. However, non-repair-related DNA synthesis (for instance, due to cell division) could potentially dilute the pool of damaged DNA and thus mimic repair. We utilized several approaches to be certain that we could specifically measure DNA repair in *C. elegans *with the QPCR assay. Cell division in adult *C. elegans *occurs only in the germline; other adult tissues are composed entirely of terminally differentiated cells [30]. Therefore, by using the *glp-1 *mutant, which is completely defective in germline proliferation at 25°C, we were able to obtain a direct measure of photoproduct repair. A second potential confounder is the existence of endoreduplication in this species. Although we cannot completely rule out an effect of endoreduplication in the *glp-1 *adults, it is unlikely to have been an important factor because postlarval endoreduplication is limited in terms of both the degree of ploidy achieved and the number of cells in which it occurs [45].

The choice of *glp-1 *mutants for the study of repair kinetics was important, because the apparent rate of nuclear and mitochondrial repair is elevated in N2 compared with *glp-1 *adults (Figure 3a). That difference cannot likely be explained by more efficient repair of UVC-induced nuclear damage in *glp-1 *than N2 nematodes, because nuclear repair rates in N2 and *glp-1 *starved L1 larvae were indistinguishable (Figure 4). Therefore, the high apparent rate of repair in N2 adults is probably the result of some combination of three mechanisms: faster kinetics of DNA repair in germ cells than in other cells; a high rate of germ cell division, and probably translesion synthesis, despite a high level of UV-induced DNA damage; or a high rate of apoptosis. DNA damage induces apoptosis in germ cells, but not somatic cells, in *C. elegans *[17]. Thus, in theory, damaged genomes in germ cells could be completely removed via apoptosis, and replaced with newly synthesized DNA via cell division, reducing the level of remaining damage beyond what repair alone could achieve. However, the lesion frequency we observed implies about 500 lesions/chromosome, which means that genome replacement would also be dependent either on germ cell specific high repair rates before replication, or on translesion synthesis (TLS). TLS is catalyzed by specialized DNA polymerases that efficiently bypass UV-induced photoproducts [46]. This is a strong possibility because *C. elegans *has homologs of the human TLS polymerase η (*polh-1 *[47]) and κ (*polk-1*), and previous evidence for a high capacity for TLS in this species exists [37].

Photoproducts also disappeared over time from mitochondrial DNA in N2 adults (Figure 3a). Because NER is not operative in mitochondria [48], this reduction in average number of lesions in mitochondrial DNA must occur through some combination of removal of damaged genomes and production of new genomes. Assuming a Poisson distribution of UV damage among mitochondrial nucleotides, at this lesion frequency (about 1.2 lesions/10 kb in N2 adults), only approximately 20% of the mitochondrial genomes (13,794 bp in *C. elegans*) in a cell are expected to be free from damage. Although most cells would have undamaged templates available for copying (about 70 copies per somatic cell, about 250 per germline nucleus, and about 18,000 per oocyte [49]), a large proportion of the total content would need to be replaced. This suggests a remarkable capacity for replacement of damaged mitochondrial genomes, and raises the interesting question of how turnover of mitochondrial DNA damage is regulated. This process must be dependent on or at least accelerated by cellular replication, because it occurred poorly or not at all in *glp-1 *adults (Figure 3b).

Repair of UVC-generated nuclear DNA damage in *glp-1 *adults was biphasic, including a rapid repair component with a t_1/2 _of about 16 hours and a much slower component evident after about 24 hours. The decline in repair after 24 hours may be attributable to slower kinetics of repair of a specific kind of lesion, tissue-specific differences in repair rates, or a nonspecific process related to poor physiologic condition. The rate of repair that we measured is comparable to that measured by Hartman and coworkers [36] using antibodies to CPDs and 6,4-photoproducts, although in our experiments repair did not appear to be saturated during the initial 24 hours after exposure (as indicated by first order kinetics). Moreover, the rate of repair we measured by QPCR in *C. elegans *is within the range of repair rates observed in human cells in culture using the same assay [20,28], although rates of repair in human cells depend significantly on the cell type. On the other hand, repair of UVC-mediated DNA damage is faster in bacteria and yeast [44,50,51].

We also examined DNA repair in *xpa-1 *starved L1 larvae. Many homologs of human DNA repair genes have been identified in cDNAs or the sequenced genome of *C. elegans *[7,8]. In some cases the involvement of those genes in DNA repair has been supported by showing that mutations or RNAi knockdown provided a genotoxin-sensitive phenotype such as accumulation of mutations or UV sensitivity [9,11,13,52-54]. However, the role of any of these genes in repair *per se *has not been directly demonstrated in *C. elegans*. *xpa-1 *mutants carry a carboxyl-terminal deletion that eliminates 80% of the gene, including the zinc finger motif and other regions that are important for DNA binding, as well as most or all of the regions homologous to the exons required for UV resistance in human cells [55]. In addition, *xpa-1 *mutants are UV-sensitive, as demonstrated by decreased viability and fertility following exposure to UV irradiation [56] (Astin J, Kuwabara P, personal communication). As expected, repair of UVC damage was not detected in *xpa-1 *starved L1 nematodes (Figure 4).

### Repair of UVC-induced DNA damage in poorly and well expressed nuclear genes

NER is the repair pathway expected to remove the great majority of UVC-induced DNA damage. In organisms from bacteria to humans, NER consists of two distinct molecular pathways [22,23]: GGR, in which lesions present in any portion of the genome are detected and removed; and TCR, in which lesions are detected and subsequently removed when they block the progression of RNA polymerase II. We expected that the same would be true in *C. elegans *based on the evolutionarily ancient nature of these two pathways, the presence of NER homologs in *C. elegans*, and the functionality of the *C. elegans *homolog of human XPA (Figure 4). The QPCR assay does not directly test damage in the transcribed and nontranscribed strands of genomic DNA, because lesions on either strand will reduce PCR amplification. However, we did find that more highly expressed genes were repaired more quickly, both in young and aging adults (Table 1). This result is consistent with the presence of GGR and TCR in *C. elegans*. It is worth noting that the difference in repair kinetics between transcribed and nontranscribed DNA strands would presumably in fact be larger than the kinetic difference between transcribed and nontranscribed genes that we measured, because only one strand of transcribed genes is transcribed and repaired by TCR. Additionally, all of our medium and high expression targets include distal portions of genes, which are less affected by TCR than proximal portions [57], and some include intergenic sequence (for example, *act-1*, *act-2*, and *act-3*) or lower expression genes (for example, *act-4*; Table 1). The presence of all three types of sequence in our high expression targets is expected to reduce the contribution of TCR to repair in those targets.

Decline of DNA repair in aging *C. elegans *and mammals

We found that repair of UVC-damaged DNA was slower in aging (6 days after L4, corresponding to 60% of the mean adult lifespan) than in young (1 day after L4, corresponding to 10% of the mean adult lifespan) *glp-1 *adults. This is the first evidence of an age-related decline in DNA repair in a whole organism. The decrease of DNA repair with age in *C. elegans *cannot be explained trivially as the result of an age-related decrease in transcription, because expression of the genomic targets in which we measured repair did not decrease with age (Table 1). Furthermore, the rate of repair in all genes, not just highly transcribed genes, decreased with age (Table 1).

Previous evidence for an age-related decline in DNA repair was obtained largely from cell culture systems. For example, decreased repair has been observed in some but not all cases in mammalian cells undergoing senescence in culture [58,59], as well as cultures of primary cells taken from older versus younger individuals [26,60-64]. Additionally, there is a general correlation between mammalian lifespan and DNA repair (for review [65]). Further support for a relationship between DNA repair and aging comes from the existence of several human diseases caused by DNA repair defects that result in shortened lifespan in affected humans as well as rodent models, despite the much shorter normal rodent lifespan [24,25,66,67]. Finally, a recent study reported that the *in vivo *repair of CPDs is decreased in the skin of old compared with that of young men, suggesting that the previous cell culture results are reflective of *in vivo *biology [27].

In terminally differentiated cells in mammals, global repair decreases, but repair of transcribed genes is maintained [68,69]. Our results indicate a similar pattern in aging *glp-1 *adult *C. elegans*, in which all cells are terminally differentiated; although repair was slowed both in highly and negligibly expressed nuclear regions (Table 1), the degree of decrease was least in the highly transcribed genes (about 30% versus about 50% in genes expressed at negligible or low/medium levels; Table 1).

The observation of decreased repair in aging nematodes is also interesting because of the current lack of support for any relationship (correlative or otherwise) between DNA repair and aging in *C. elegans*. *C. elegans *researchers have identified dozens of genes that, when mutated or knocked down, result in up to several-fold longer lifespans [6,70,71]. Some of the pathways that these genes belong to also affect lifespan in mammals (for example, see Holzenberger and coworkers [72]). Despite the large number of genes identified by RNAi knockdown and mutation screens, DNA repair genes have not been identified as involved in altering lifespan in the nematode. However, finding a DNA repair gene for which a decrease in function increases lifespan seems unlikely, and screens rarely attempt to identify mutations that cause accelerated aging because a shortened lifespan can be caused by many factors that are not necessarily related to normal aging.

Previous studies with specific genes in *C. elegans *have produced mixed results regarding a possible relationship between DNA repair and aging. In keeping with mammalian systems [73], RNAi knockdown of a *C. elegans *Werner syndrome homolog did decrease lifespan [11]. Furthermore, an early report detected an age-dependent increase in single strand breaks, and an associated decrease in transcriptional capacity, in genomic DNA from *C. elegans *[74]. Nonetheless, to date, experiments designed to test more directly a possible connection between DNA repair and aging in *C. elegans *have found no association. Hartman and coworkers [18] found no relationship between UVC or ionizing radiation sensitivity and lifespan among four strains with variable lifespans. Johnson and Hartman [75] also found that only one of seven previously identified UVC-sensitive mutants had a short lifespan; furthermore, they found that the effect of ^137^Cs radiation on the lifespan of the mutants and wild-type *C. elegans *was generally similar. These studies did not directly measure DNA repair, however, and could include only a small subset of long-lived mutants and DNA repair genes. Although our experiments did not test whether decreased NER can cause aging, they do clearly establish that NER decreases in aging nematodes. Finally, it is important to point out that although UV damage is probably of little ecologic relevance to soil-dwelling *C. elegans*, NER is surely of great biologic relevance. NER is highly conserved throughout evolution and repairs a wide range of structurally diverse types of DNA damage, including fungal and plant secondary compounds [23].

### Possible mechanisms of decreased DNA repair in aging *C. elegans*

Why does repair of UV-mediated DNA damage decrease in aging nematodes? We address three possibilities below: transcriptional downregulation of NER genes; altered protein function or trafficking; and a decrease in energy production.

Age-dependent downregulation of NER and NER-related genes in adult nematodes does not appear to be responsible (Table 2), although the general decrease in NER gene expression from the embryonic to the adult stages may account at least in part for the decrease in repair rate between those stages previously reported by Hartman and coworkers [36]. In fact, we detected no significant changes in the expression of any identified DNA repair gene (not just NER genes) between 1-day and 6-day adults (GEO accession number GSE4766).

Another possibility is that nontranscriptional changes are responsible for the decrease in repair. For example, Szczesny and coworkers [76] showed that although the transcription and activity of mitochondrial-targeted base excision repair genes were increased in hepatic tissue from old rodents, repair was probably decreased because of a failure of mitochondrial import of the proteins.

A related possibility that might be detectable by gene expression analysis is that DNA repair rates decreased in aging adults because cellular functions necessary to support repair were impaired. Analysis of Gene Ontologies that were altered in aging vs young nematodes revealed several intriguing differences (Table 3). Our results suggest a decrease in many processes that are fundamental to homeostasis, including ion transport, catalytic activity, and energy production.

Although additional experiments will be required to test hypotheses generated by our gene expression data, one result is of particular interest because of additional supporting data. Many genes encoding proteins that are involved in the TCA cycle and oxidative phosphorylation, necessary for ATP synthesis, were expressed at lower levels in aging adults. This was true of both nuclear and mitochondrial encoded proteins, and was also coincident with a decrease in mitochondrial DNA copy number in aging *glp-1 *adults (data not shown). There are no transcriptional data to support the hypothesis that there was a compensatory increase in glycolytic energy production (Table 3). A decrease in transcripts for proteins required for energy production was previously observed by McCarroll and coworkers [77] in aging *C. elegans *as well as *Drosophila melanogaster*. These decreases in mRNA levels may be functionally significant, because metabolic capacity and ATP content decrease with age in *C. elegans *[78-80], as in humans [81]. The preceding studies and others [82] indicate that total cytoplasmic ATP is normally in the 2-4 mmol/L range in young adult *C. elegans*, but decreases by fivefold to tenfold with age. Although NER requires ATP, no precise *in vivo *measurements of the dependence of NER on ATP concentration have been made. However, *in vitro *assays suggest that such a decrease in ATP could significantly affect the rate of NER [83]. These findings suggest the hypothesis that reduced repair is due in part to reduced available energy. Such a mechanism would be expected to reduce repair of both transcribed and nontranscribed genes, which is in keeping with our data.

## Conclusion

Repair of UVC-induced lesions in *C. elegans *is similar to the analogous process in mammals. Additionally, we found that repair decreases with age in *C. elegans*. Previous studies have suggested that DNA repair capacity may be related to age, but it has been difficult to test this hypothesis in an intact organism. Gene expression profiling suggested that age-related decreased mitochondrial function and ATP production mediate the concurrent decrease in repair capacity. The use of the QPCR assay with *C. elegans *permits the study of the formation and repair of many types of DNA damage *in vivo *in a multicellular organism, and promises to further increase the utility of this organism for the study of processes such as aging, apoptosis, neurodegeneration, and carcinogenesis.

## Materials and methods

### *C. elegans *culture

Populations of *C. elegans *were maintained on K agar plates seeded with OP50 bacteria [84]. Semi-synchronized populations of nematodes were obtained by bleach-sodium hydroxide isolation of eggs [85]. L1 growth-arrested (starved) larvae were obtained by hatching eggs in M9 buffer overnight with shaking [86]. Fed L1 larvae were obtained by allowing semi-synchronized egg batches to feed for 12 hours at 20°C before exposure. Homogenous populations of dauer larvae were obtained by placing 2000 to 3000 growth-arrested L1 larvae on 100 mm lightly seeded plates at room temperature (about 23°C), such that sufficient food was provided to allow exit from the L1 stage, but preclude normal (nondauer) development. Starved L1 larvae were used immediately (<24 hours after hatch); dauer larvae were used 24 to 48 hours after reaching the dauer stage. For studies with 6-day *glp-1 *adults (6 days after L4 molt), nematodes were transferred to fresh seeded K agar plates daily. All transfers were by washing off and rinsing (after centrifugation at 2000 *g *for 2 min, or after settling by gravity in the case of adults) in K medium [87].

The survival curve for *glp-1 *adults was obtained at 25°C. One-day-old adults (1 day after L4 molt) are described in this report as 'young' adults, whereas 6-day-old adults (6 days after L4 molt) are described as 'aging' adults. One day after L4 molt was counted as 'day 1'; populations were maintained at 25°C from hatch. A total of eight replicate plates were monitored; three separate (in time) experiments were carried out. Plates had between 23 and 32 noncensored nematodes each, for a total '*n*' of 213. Censored worms were those that crawled onto the sides of the petri dishes and desiccated, were lost during transfer, or disappeared but were not observed to be dead. Nematodes were transferred by washing on a daily basis during the first 7 to 8 days and every 1 to 3 days thereafter.

Strains JK1107 (referred to in this manuscript as '*glp-1*') and RB864 ('*xpa-1*') were obtained from the *Caenorhabditis *Genetics Center (University of Minnesota). *xpa-1 *has recently been identified as allelic to the previously characterized [56] UV-sensitive *C. elegans *strain *rad-3(mn157) *(Astin J, Kuwabara P, personal communication).

### UVC exposures

UV radiation exposures were done in a CL-1000 Ultraviolet Crosslinker (UVP, Upland, CA, USA) with an emission peak at 254 nm (referred to in this manuscript as 'UVC'); no differences were observed in initial comparisons of dose-response curves after UVC exposure by the Crosslinker or by a UVC lamp monitored with a UV meter (data not shown). Nematodes at all life stages were rinsed three times with K medium and allowed to settle for 10 min between rinses in order to remove residual bacteria; nonadult life stages were centrifuged between rinses. This time period also permitted elimination of the majority of ingested bacteria except in the case of 6-day-old adults, which did not clear their guts even after hours of incubation in clean K medium. After rinsing, nematodes were aliquoted onto nonseeded K agar plates in 500 μl volumes and spread evenly using a bent glass rod. The nematodes were plated in sufficiently small numbers that shielding was inconsequential (few or no nematodes were observed to overlay each other). Plates were placed in the Crosslinker for exposure within 5 min of plating to minimize evaporation of the medium and clumping of the nematodes. After exposure, nematodes were rinsed off the plates, centrifuged, and frozen within 10 to 15 min (except for repair experiments). Worms were frozen by dripping pelleted worms suspended in about 500 μl K medium into liquid nitrogen, and stored at -80°C.

### DNA and RNA extraction

For nucleic acid extraction, pellets containing 2,000 to 5,000 nematodes were ground into fine powder with a liquid nitrogen-cooled mortar and pestle [88] and then extracted using either an RNeasy kit (Qiagen, Valencia, CA, USA) or a Genomic Tips kit (Qiagen; following the protocol for extraction of genomic DNA from cells in culture). Alternatives to the liquid nitrogen grinding procedure were attempted for DNA extraction (including homogenization, bead beating, three rounds of freeze-thaw, and simple incubation with the Genomic Tips digestion buffer from Qiagen, proteinase K and RNase A), but all resulted in the extraction of degraded genomic DNA. The integrity of genomic DNA after different extraction methods was evaluated by examination of high-molecular-weight genomic DNA using agarose gel electrophoresis and comparison of amplification of long PCR products from equal amounts of template (QPCR; described below). RNA was quantified with a NanoDrop Fluorospectrometer (NanoDrop Technologies, Wilmington, DE, USA) and analyzed for integrity with a BioAnalyzer (Agilent Technologies, Santa Clara, CA, USA). DNA quantity was measured before QPCR using PicoGreen dye (Invitrogen Corporation, Carlsbad, CA, USA), as described previously [21].

### Quantitative PCR (QPCR)

We developed primers and optimized PCR conditions to adapt our QPCR methodology [21] to *C. elegans*. Primers, product size, typical cycle number, and annealing temperature are listed for all QPCR targets in Additional data file 1. Note that the nuclear gene targets listed are in some cases the only identified gene amplified by the primers listed, but in other cases they constitute only the majority (not the entirety) of the amplicon. Additional detail on the nuclear genomic regions amplified is presented in Table 1. Different nuclear targets were chosen to include genes that we expected to be transcribed at negligible, medium, or high levels in *glp-1 *adults, based initially on available expression data including the Kohara cDNA library [89], the Kohara *in situ *hybridization database [90], and published gene expression experiments [91,92]. Our expectations regarding the general level of expression of specific genes in adults were confirmed by our gene expression analysis (Table 1). The mitochondrial large product amplifies the majority of the mitochondrial genome (10.9 kb out of 13.8 total), excluding the AT-rich and adjoining regions [93]. The small mitochondrial and nuclear products are located at one extreme of the large mitochondrial and the polymerase epsilon targets, respectively.

All primer pairs produce unique products (assessed by agarose gel electrophoresis with ethidium bromide staining) using *C. elegans *genomic DNA as a template under the conditions listed, and all QPCR products were tested for identity by size before and after restriction enzyme digestion (data not shown). BLAST searches for nuclear sequences similar to the small mitochondrial product or primers failed to identify any sequences of significant similarity. Additionally, all primer pairs were tested at 35 cycles with 10 ng purified OP50 bacterial genomic DNA as a template. Only the *unc-44 *primers amplified any product; the bands produced were shorter than that produced with nematode DNA. However, no detectable band was produced at the cycle number used for amplification of nematode DNA. For experiments in which the *unc-44 *primers were used (Table 1), the PCR product was subjected to agarose gel electrophoresis as well as quantification by plate reader, and no bands indicative of amplification of OP50 genomic DNA were observed.

The 50 μl QPCR reaction mixtures contained the following: 9.6 μl sterile de-ionized water, 15 μl 3.3× r*Tth *XL DNA polymerase buffer, 5 μl 1 mg/ml bovine serum albumin, 4 μl dNTPs (2.5 mmol/l of each), 2.4 μl 25 mM MgO(Ac)_2_, 2 μl of each primer, and 5 μl of 2 ng/μl genomic DNA template. All primers were used at 10 μmol/l except the small mitochondrial primers, which were used at 7.5 μmol/l (final concentrations 0.4 and 0.3 μmol/l). r*Tth *XL DNA polymerase was diluted in 1× buffer, and 5 μl was added in a hot start procedure. The 3.3× buffer, MgO(Ac)_2_, and r*Tth *XL DNA polymerase were from the GeneAmp XL PCR kit (Applied Biosystems, Foster City, CA, USA). The cycling conditions for the small mitochondrial and small nuclear targets are as follows: 75°C for 2 min; 94°C for 1 min; 94°C for 15 s, 63°C for 45 s, and 72°C for 30s (repeated as indicated in Table 1); and 72°C for 5 min. The cycling conditions for the large mitochondrial and small nuclear targets are: 75°C for 2 min; 94°C for 1 min; 94°C for 15 s and 66/68/70°C for 12 min (repeated as indicated in Table 1); and 72°C for 10 min.

We recently reported a detailed protocol for QPCR [21] that should be read by researchers who are interested in using the technique, because QPCR requires quality control steps beyond those normally employed in PCR reactions. The assay as applied to *C. elegans *is generally similar. Exceptions are the conditions listed above and in Additional data file 1, and as follows. First, a small nuclear, as well as a small mitochondrial, target was developed. The small mitochondrial target is necessary in all organisms, because mitochondrial DNA copy number per cell is variable. A small nuclear target is not necessary with most organisms, because exact amounts of starting DNA template are used in the QPCR assay. However, it was necessary to use a small nuclear normalization product for *C. elegans*. Because it was not possible to eliminate bacteria from the guts of 6-day-old worms, some of the DNA recovered from the extraction process was bacterial rather than nematode in origin, making addition of identical amounts of starting nematode genomic DNA template impossible. Second, hot start was used for all QPCR reactions, not just those of the larger products. Third, the r*Tth *XL DNA polymerase was diluted in 1× PCR buffer rather than water, before hot start. Additional procedural details are available upon request.

### Analysis of repair

DNA repair was assessed by waiting fixed amounts of time after UVC exposure before freezing the nematodes. In most cases, nematodes were not fed after exposure because of the short time courses involved, and in the case of studies with L1 larvae to avoid the confounder of cell division mediated by L1 exit. In the case of the 72 hour repair time courses with adult nematodes (Figure 4), two experiments were performed in the presence of food, and the other experiments were performed in the absence of food. No differences were observed. Repair was measured in the polymerase epsilon target for Figures 3 to 5. Repair in adults was assessed at 25°C, the temperature at which *glp-1 *adults do not produce eggs. Repair in L1 larvae was assessed at 20°C.

### Analysis of genomic deletions and mRNA expression by PCR

After initial characterization of repair indicated that the *xpa-1 *strain RB864 was repair deficient (Figure 5), we confirmed the presence of the deletion by PCR using previously developed primers [94], outcrossed three times with N2 wild-type nematodes, and repeated the repair studies (Figure 5). We also designed RT-PCR primers to quantify mRNA of Y50D7A.2 (forward: 5'-GAGCACTTCAGGAGCTACAAAGC-3'; and reverse: 5'-GCCCAAACATCTCCGTTATCA-3'; product size 415 nucleotides) and *csb-1 *(forward: 5'-GCCGAGAAGGGAATCA AATG-3'; and reverse: 5'-CTTGTTCTTCATCCGTTTCTTGG-3'; product size 525 nucleotides). The primers for *csb-1*, *xpc-1*, and Y50D7A.2 were designed to include exon-exon junctions to preclude the amplification of any contaminating DNA. mRNA expression levels were normalized to actin, which was measured using previously published primers [95]. First-strand cDNA was generated using oligodT primers and SuperScript II Reverse Transcriptase (Invitrogen), according to the supplied protocol, and subsequent PCR amplifications were performed with hot start and under cycle-optimized conditions with Taq DNA Polymerase (Invitrogen).

### Statistical analysis

DNA lesion frequency data were compared by analysis of variance followed, when appropriate, by Fisher's protected least significant difference *post hoc *analysis. Statistical analysis of gene expression data was performed as described below.

### Gene expression analysis

Gene expression data for *glp-1 *nematodes were obtained by comparing mRNA expression levels in replicate pools of 2,000 to 3,000 mixed-stage embryos, young (1-day) adults, and aging (6-day) adults. Mixed-stage embryos were obtained as described above, and semi-synchronized populations of young and aging adults uncontaminated by developing oocytes and associated transcripts were obtained by transferring mixed-stage embryos to 25°C. Three replicate pools of embryo and young adult samples were analyzed, but only two replicate pools of 6-day adults were analyzed because of low RNA recovery. RNA samples were extracted and analyzed for quality as described above.

Gene expression analysis was conducted using *C. elegans *Genome Genechip^® ^arrays (Affymetrix, Santa Clara, CA, USA). Starting with 1 μg total RNA, biotin-labeled cRNA was produced using the Affymetrix 3' Amplification One-Cycle Target labeling kit, in accordance with the manufacturer's protocol. For each array, 15 μg amplified cRNAs were fragmented and hybridized to the array for 16 hours in a rotating hybridization oven using the Affymetrix Eukaryotic Target Hybridization Controls and protocol. Slides were stained and washed as indicated in the Antibody Amplification Stain for Eukaryotic Targets protocol, using the Affymetrix Fluidics Station FS450. Arrays were then scanned with an Affymetrix Scanner 3000. Data were obtained using the Genechip^® ^Operating Software (version 1.2.0.037). The resulting files were loaded in the Rosetta Resolver system (version 5.1) and data were analyzed for statistical significance [96,97]. Microarray data and Rosetta Resolver-derived *p *values for all pair-wise comparisons have been deposited in the National Center for Biotechnology Information's GEO [98] and are accessible through GEO series accession number GSE4766.

Analysis of gene expression data was performed using a variety of software, including GeneSpring (Agilent), GOMiner [32], and Cytoscape [31]. Data transformation procedures for GeneSpring were as follows: set measurements less than 0.01 to 0.01; per chip, normalize to 50th percentile; and per gene, normalize to the mean of the young values. For GOMiner analyses, only genes identified as significantly differentially expressed (*P *< 0.001) between young and aging adults by Rosetta Resolver were included. In addition, expression profiles for genes represented by multiple probes were condensed by identifying and using only median values. GOMiner and Cytoscape analyses were used only to compare data from young and aging adults. The Cytoscape analysis is based on a combined interactome with 4,669 nodes and 23,785 edges, consisting of interactions downloaded from the Biomolecular Interaction Network Database (BIND) on 28 March 2006 (3,228 nodes, 5602 edges), and the interactome (2,254 nodes, 18,183 edges) reported by Zhong and Sternberg [99]. BIND is a curated database of biomolecular (largely protein-protein and protein-DNA) interactions, whereas the Zhong and Sternberg interactome is made up of computationally derived genetic interactions. The jActiveModules plugin was used to identify the most significantly altered subnetworks of connected genes, assign Z scores, and calculate *p *values, as described previously [31,100]. Z scores and *p *values were calculated with a depth of one neighbor and 10,000 Monte Carlo iterations. For Cytoscape analysis, important GO terms were identified as the following: the GO terms associated with the top 20 nodes (genes) presented in Figure 7 and Additional data file 4 (part A); the GO terms associated with the top 20 Active Modules analyzed at a depth of two edges from each central node, presented in Additional data file 4(part B; identified by the BiNGO plugin [33]); and the GO terms identified by analysis of the entire dataset using the BiNGO plugin, presented in Additional data file 5.

## Additional data files

The following additional data are available with the online version of this paper. Additional data file 1 contains the specifics of the *C. elegans *amplicons used for QPCR. Additional data file 2 shows photographs of young and aging *glp-1 *adults raised at 25°C. Additional data file 3 includes lists of altered Gene Ontologies identified by GOMiner when comparing young with aging *glp-1 *adult *C. elegans *raised at 25°C. Additional data file 4 identifies and presents BiNGO analysis of the 20 top-scoring active modules representing altered gene neighborhoods, when comparing young with aging *glp-1 *adult *C. elegans *raised at 25°C. Additional data file 5 provides results of BiNGO analysis of the entire dataset comparing young with aging *glp-1 *adult *C. elegans *raised at 25°C.

## Supplementary Material

Additional data file 1 Provided are the specifics of the *C. elegans *amplicons used for QPCR (genomic target amplified, size of QPCR target, typical cycle number for quantitative analysis, forward and reverse primers, and annealing temperature). Click here for file

Additional data file 2 Shown are photographs of young (1-day-old) and aging (6-day-old) *glp-1 *adults raised at 25°C, at 10× and 20× magnification. Hollow arrows point to tails without blisters, and solid arrows point to blisters. Click here for file

Additional data file 3 Provided are lists of altered Gene Ontologies (cellular component, molecular function, and biologic process) identified by GOMiner, when comparing young (1-day) with aging (6-day) *glp-1 *adult *C. elegans *raised at 25°C. Click here for file

Additional data file 4 Part A lists the 20 top-scoring active modules representing altered gene neighborhoods identified by Cytoscape, when comparing young (1-day) with aging (6-day) *glp-1 *adult *C. elegans *raised at 25°C. Part B lists the results of BiNGO analysis of the 20 top-scoring active modules representing altered gene neighborhoods identified by Cytoscape, when comparing young (1-day) with aging (6-day) *glp-1 *adult *C. elegans *raised at 25°C. Click here for file

Additional data file 5 Presented are the results of BiNGO analysis of the entire dataset comparing young (1-day) with aging (6-day) *glp-1 *adult *C. elegans *raised at 25°C. Click here for file
