# Supplementary figures and images for: Decline of nucleotide excision repair capacity in aging Caenorhabditis elegans
Source: Genome Biol. 2007 May 1;8(5):R70. doi: 10.1186/gb-2007-8-5-r70 (PMC1929140; doi:10.1186/gb-2007-8-5-r70)

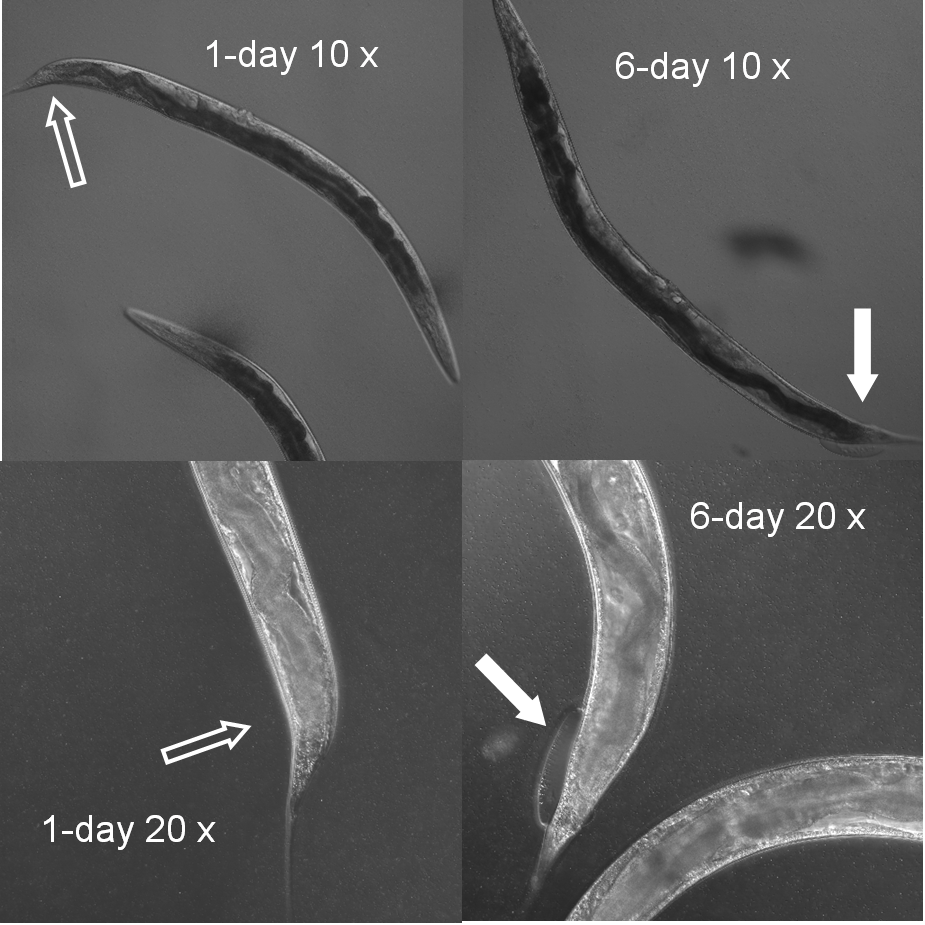

Supplement: Additional data file 2 — Shown are photographs of young (1-day-old) and aging (6-day-old) glp-1 adults raised at 25°C, at 10× and 20× magnification. Hollow arrows point to tails without blisters, and solid arrows point to blisters. [file gb-2007-8-5-r70-S2.tiff]
